# Immunomodulatory matrix-bound nanovesicles mitigate acute and chronic pristane-induced rheumatoid arthritis

**DOI:** 10.1038/s41536-022-00208-9

**Published:** 2022-02-02

**Authors:** Raphael J. Crum, Kelsey Hall, Catalina Pineda Molina, George S. Hussey, Emma Graham, Hongshuai Li, Stephen F. Badylak

**Affiliations:** 1 McGowan Institute for Regenerative Medicine, University of Pittsburgh, 450 Technology Drive, Suite 300, Pittsburgh, PA 15219 USA; 2 Department of Surgery, School of Medicine, University of Pittsburgh, University of Pittsburgh Medical Center Presbyterian Hospital, 200 Lothrop Street, Pittsburgh, PA 15213 USA; 3 ECM Therapeutics, Inc., 118 Marshall Dr., Warrendale, PA 15086 USA; 4 Musculoskeletal Growth and Regeneration Laboratory, Department of Orthopaedic Surgery, University of Pittsburgh, 450 Technology Drive, Suite 206, Pittsburgh, PA 15219 USA; 5 Department of Orthopedics and Rehabilitation, University of Iowa, 25 Grand Ave, Iowa City, IA 52246 USA; 6 Department of Bioengineering, University of Pittsburgh, 3700 O’Hara Street, Pittsburgh, PA 15261 USA

**Keywords:** Autoimmunity, Regenerative medicine, Tissue engineering, Preclinical research

## Abstract

Rheumatoid arthritis (RA) is an autoimmune disease characterized by chronic inflammation and destruction of synovial joints affecting ~7.5 million people worldwide. Disease pathology is driven by an imbalance in the ratio of pro-inflammatory vs. anti-inflammatory immune cells, especially macrophages. Modulation of macrophage phenotype, specifically an M1 to M2, pro- to anti-inflammatory transition, can be induced by biologic scaffold materials composed of extracellular matrix (ECM). The ECM-based immunomodulatory effect is thought to be mediated in part through recently identified matrix-bound nanovesicles (MBV) embedded within ECM. Isolated MBV was delivered via intravenous (i.v.) or peri-articular (p.a.) injection to rats with pristane-induced arthritis (PIA). The results of MBV administration were compared to intraperitoneal (i.p.) administration of methotrexate (MTX), the clinical standard of care. Relative to the diseased animals, i.p. MTX, i.v. MBV, and p.a. MBV reduced arthritis scores in both acute and chronic pristane-induced arthritis, decreased synovial inflammation, decreased adverse joint remodeling, and reduced the ratio of synovial and splenic M1 to M2 macrophages (*p* < 0.05). Both p.a. and i.v. MBV reduced the serum concentration of RA and PIA biomarkers CXCL10 and MCP-3 in the acute and chronic phases of disease (*p* < 0.05). Flow-cytometry revealed the presence of a systemic CD43hi/His48lo/CD206+, immunoregulatory monocyte population unique to p.a. and i.v. MBV treatment associated with disease resolution. The results show that the therapeutic efficacy of MBV is equal to that of MTX for the management of acute and chronic pristane-induced arthritis and, further, this effect is associated with modulation of local synovial macrophages and systemic myeloid populations.

## Introduction

The extracellular matrix (ECM) of all tissues and organs represents the secreted product of resident cells and consists of a complex mixture of structural and bio-active molecules; thus the ECM comprises an ideal microenvironment for cells in the healthy state^1,2^. The ECM has a strong influence upon the behavior and phenotype of tissue-resident cells, a process referred to as dynamic reciprocity^3^. Cells of the immune system, such as tissue-resident macrophages, circulating monocytes, and lymphocytes are no exception to the influence of ECM. ECM derived from healthy tissues and configured into surgical mesh materials, topical powders, or hydrogels, has been shown to promote an anti-inflammatory, regulatory macrophage phenotype^4–15^.

The components of ECM that mediate an anti-inflammatory macrophage phenotype are not fully understood, but it has been shown that this macrophage phenotype transition can be induced by exposure to the degradation products of acellular biologic scaffold materials composed of mammalian ECM^4–7,10–13,16–19^, including recently characterized, matrix-bound nanovesicles (MBV)^7,9,20^. MBV are a distinct class of extracellular vesicles with a characteristic lipid membrane composition, protein cargo, and miRNA cargo that differs from that of fluid-phase exosomes^7,20,21^. In vitro studies have shown that MBV, independent of their parent ECM, are able to recapitulate the effects of whole ECM upon macrophage phenotype^7,20^. Specifically, MBV induce macrophage gene, protein, and cell surface marker expression patterns associated with the functional capacities of anti-inflammatory, pro-remodeling M2 macrophages^7,20^. Additionally, MBV increases phagocytic activity and antimicrobial activity representative of an M2 macrophage phenotype which is consistent with previous studies that have investigated the effects of ECM bioscaffolds on macrophage phenotype^4–6,10–13,16^. The M1 to M2 immunomodulation observed with MBV and ECM bioscaffolds has considerable potential for the clinical translation since many disease pathologies, including autoimmune diseases like rheumatoid arthritis (RA), are driven by a fundamental disequilibrium of this M1:M2 ratio^22–24^.

The immune system is comprised of a diverse collection of cells and signaling molecules that maintain tissue homeostasis in a state of healthy physiology. Autoimmune diseases are characterized by a dysregulation of the immune system and a pro-inflammatory response directed against self-antigens and tissues with associated tissue damage^25–28^. RA is an autoimmune disease characterized by synovial joint invasions of pro-inflammatory immune cells, such as neutrophils and macrophages, that are directly involved in the disease pathogenesis and progression^24,27,29–33^. Specifically, the activation of macrophages toward an M1, pro-inflammatory phenotype strongly contributes to RA disease flareup, whereas an M2, anti-inflammatory phenotype contributes to disease remission^23,24,34^. Patients afflicted with RA present to the clinic with an increased ratio (as high as 30-fold) of M1 to M2 macrophages in their joint synovium and synovial fluid^22,35^. While it is known that an M1:M2 disequilibrium contributes to RA disease progression, there are no therapeutics presently available that specifically modulate macrophage phenotype to promote disease remission through an M2, anti-inflammatory phenotype. Thus, there is a clear unmet need for developing approaches to modulate rather than suppress the pro-inflammatory immune response for the treatment of autoimmune diseases such as RA.

The evidence supporting ECM- and MBV-mediated immunomodulation of macrophage phenotype, combined with the clinical evidence of pro-inflammatory M1 macrophages as a key mediator of RA, provides the premise of the present research^31,35,36^. Using the pristane-induced, pre-clinical rat model of RA, it was hypothesized that MBV would reduce inflammatory arthritis disease development, decrease synovial inflammatory cell infiltration, prevent adverse cartilage remodeling, modulate synovial and systemic macrophage populations from a pro-inflammatory M1 phenotype towards an anti-inflammatory M2 phenotype, and thus promote disease resolution.

## Results

### MBV delivered locally and systemically prevent acute and chronic pristane-induced arthritis with comparable efficacy to methotrexate

**Fig. 1:**
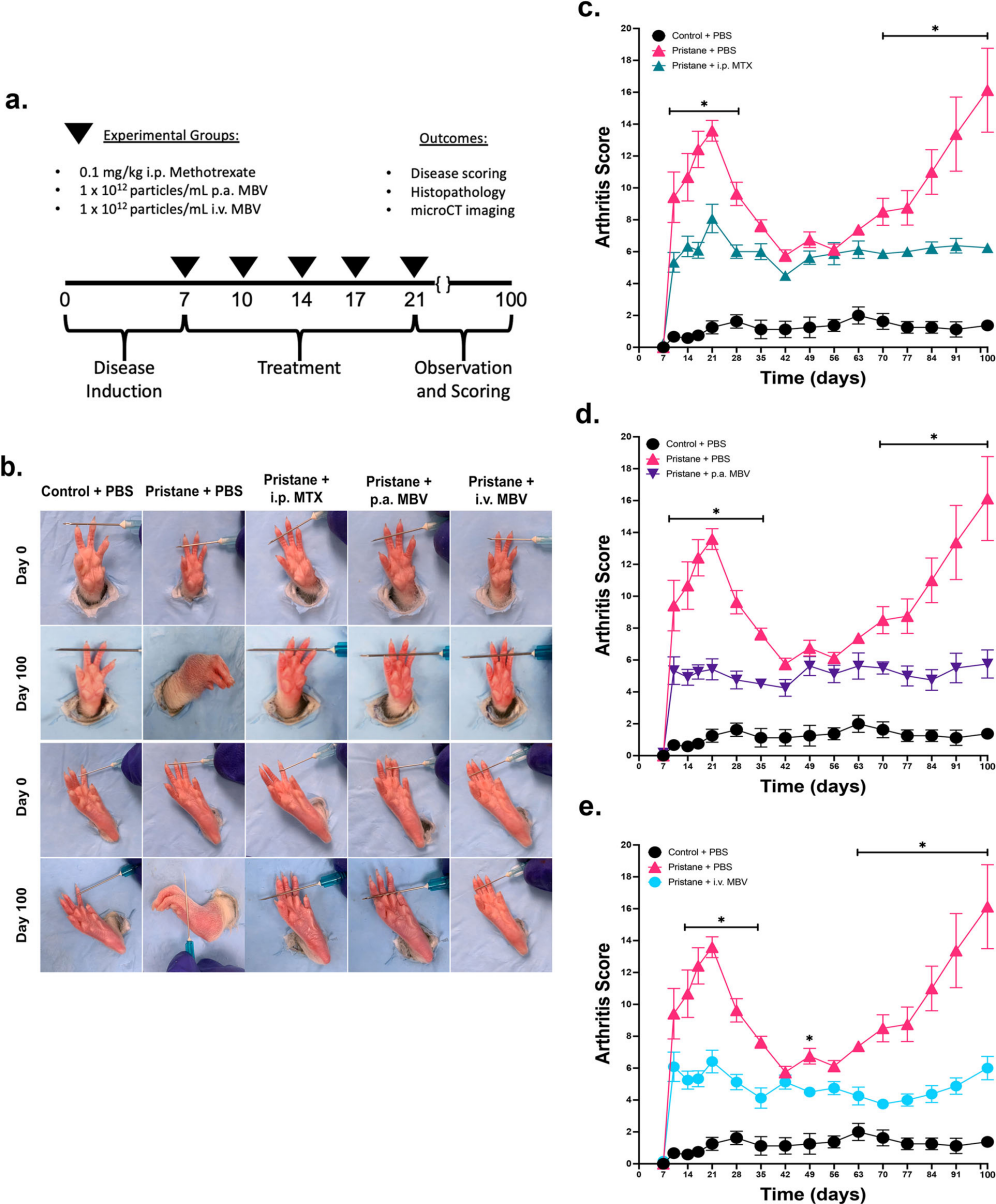
Local and systemic administration of MBV significantly reduces both acute and chronic pristane-induced arthritis disease severity. **a** Experimental design and treatment regimen. **b** Representative images of forepaws and hind paws for each group at day 0 and day 100. Evident edema, erythema, and distortion of the forepaws and hind paws were evident in the Pristane + PBS group but not in the remaining treatment groups. **c** I.p. methotrexate significantly reduces disease scoring between days 10–28 and days 70–100 compared to Pristane + PBS (*p* < .05) **d** P.a. MBV significantly reduces disease scoring between days 10–35 and days 70–100 compared to Pristane + PBS (*p* < 0.05). **e** I.v. MBV significantly reduce disease scoring between days 14–35 and days 63–100 compared to Pristane + PBS (*p* < 0.05). All values represented in panels **c**–**e** are mean ± s.e.m., *n* = 12 for days 7–28, and *n* = 8 days 28–100.

Animals were treated with methotrexate (MTX) or MBV to determine their comparative therapeutic efficacy for pristane-induced RA. MBV were delivered either to the plantar and volar surfaces of rat paws (peri-articular, p.a.) or intravenously (i.v.). MTX was delivered by intraperitoneal (i.p.) injection. In the acute phase of disease, designated as days 0–42, visual disease severity in the vehicle-treated diseased animals (Pristane + i.v. PBS) peaked at day 21, with a peak disease score of 13.6 ± 0.7. MTX treatment of diseased animals (Pristane + i.p. MTX) reduced disease severity at days 10, 14, 17, 21, and 28, with a peak disease score at day 21 (8.1 ± 0.9) (Fig. 1c, *p* < 0.05). The local, p.a. administration (Pristane + p.a. MBV) reduced disease severity at days 10, 14, 17, 21, 28, and 35, with a peak disease score of 5.5 ± 0.7 at day 21 (Fig. 1d., *p* < 0.05). The systemic, i.v. administration (Pristane + i.v. MBV) reduced disease severity at days 14, 17, 21, 28, and 35, with a peak disease score of 6.5 ± 0.7 at day 21 (Fig. 1e, *p* < 0.05). There were no significant differences among the three different treatment groups during the acute phase (*p* > 0.05); all three treatment groups were different than the disease-free, Control + PBS group (*p* < 0.05). In summary, i.p. MTX, p.a. MBV, and i.v. MBV were equally effective in reducing pristane-induced RA disease severity in the acute phase of the disease.

Since RA is a disease of a chronic relapsing-remitting phenotype, animals were observed after the acute phase to determine the long-term effect of MBV administration on chronic disease development. After day 28, and through the completion of the study at day 100, no additional MTX or MBV treatments were administered. At the beginning of the chronic phase (day 42), disease severity subsided and there was no difference among the following groups from days 42–56 (*p* > 0.05): Pristane + PBS; Pristane + i.p. MTX; Pristane + p.a. MBV; and Pristane + i.v. MBV. At day 70, the Pristane + PBS group developed a disease flare-up that continued to progress until day 100, at which time the final disease severity score was 16.1 ± 2.6. In contrast, the Pristane + MTX, +p.a. MBV, and +i.v. MBV groups did not develop this upward trend in disease severity score through day 100. The administration of MTX, p.a. MBV, and i.v. MBV resulted in a significant decrease in disease severity from days 70 to 100 for MTX (Fig. 1c., *p* < 0.05), from days 70 to 100 for p.a. MBV (Fig. 1d, *p* < 0.05), and from days 63 to 100 for i.v. MBV (Fig. 1e, *p* < 0.05). All three treatments prevented a relapse in disease severity at day 100 and there was no difference in disease scores among the three treatment groups (*p* > 0.05).

### MBV prevent adverse bone remodeling and joint destruction in the pristane-induced RA model

**Fig. 2:**
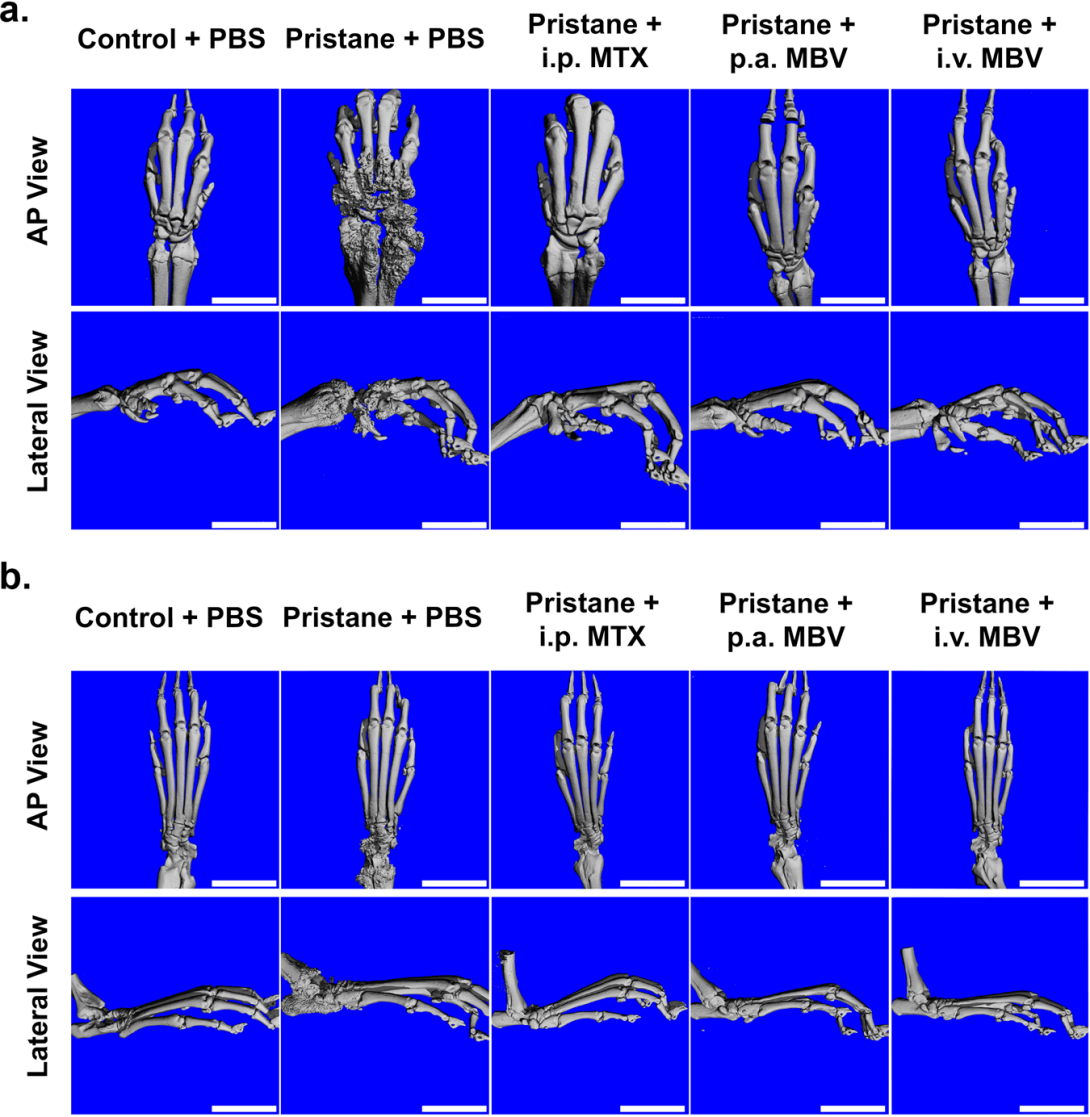
Systemic and local administration of MBV prevents adverse bone remodeling in chronic pristane-induced arthritis by microCT imaging. **a** Representative microCT images of forepaws at day 100. Substantial bone remodeling and damage are seen in the pristane + PBS group throughout the proximal bones of the forepaw while all three treatment groups show minimal to absent changes in bone morphology. Scale bar = 10 mm. **b** Representative microCT images of hind paws at day 100. Substantial bone remodeling and damage is seen in the pristane + PBS group localized to the tibiotalar joint while all three treatment groups show minimal to absent changes in bone morphology, especially in the region of the tibiotalar joint. Scale bar = 10 mm. Both panels a. and b. include both anterior–posterior (AP) and lateral views.

Pristane injection induced substantial subchondral bone damage, joint degeneration, and features of erosive arthritis as shown by microCT imaging and 3-D reconstruction of the joints (Fig. 2, Pristane + PBS). In the forepaws of untreated animals, the damage was present in the articulation of the radius and ulna with the carpal bones with lesser changes in the interphalangeal joints and carpo-phalangeal joints (Fig. 2a). In the hind paws, degenerative changes were present primarily at the articulation of the tibia and talus, as evident by joint fusion and the absence of a well-defined joint on microCT (Fig. 2b). In forepaws and hind paws, i.p. MTX, p.a. MBV, and i.v. MBV administration substantially mitigated the destructive changes in bone when compared to vehicle control. Compared to the pristane + PBS group, all three treatment groups substantially reduced qualitative bone damage and joint degeneration in the forepaws and hind paws (Fig. 2).

### Local and systemic administration of MBV decrease pro-inflammatory serum chemokines associated with clinical RA

Serum concentrations of the chemokines CXCL10 and MCP-3 are increased and positively correlated with RA disease severity in human patients as well as in pre-clinical animal models of RA^26,37–39^. These chemokines are responsible for driving both peripheral monocyte and lymphocyte recruitment to the joint tissue; together these chemokines are crucial in facilitating acute and chronic inflammation in RA^26,37–40^. Serum CXCL10 and MCP-3 were assessed throughout the course of the present study.

**Fig. 3:**
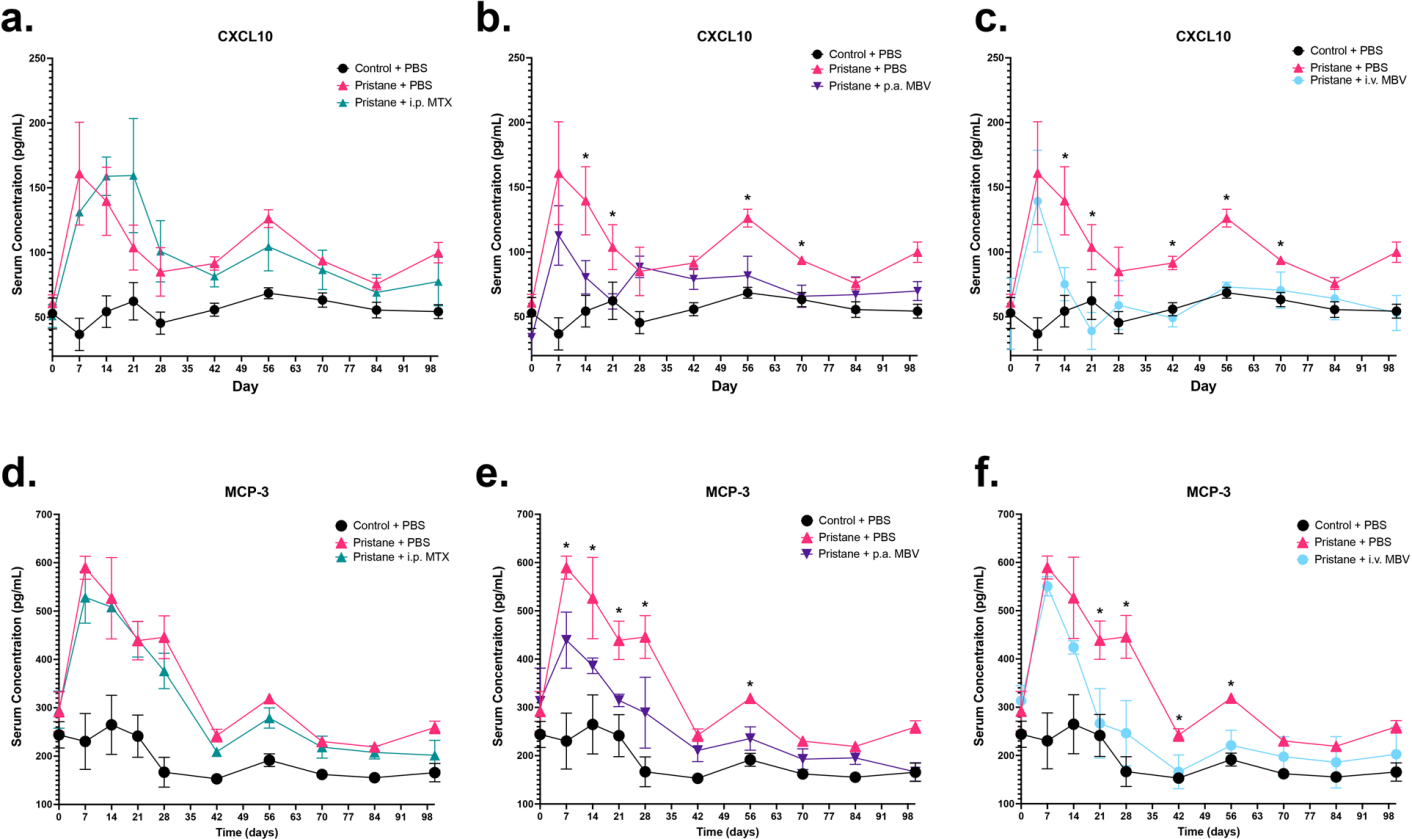
Local and systemic administration of MBV decrease the concentration of RA-associated, pro-inflammatory chemokines CXCL10, and MCP-3 in both the acute and chronic phases of disease. **a** Serum concentration of CXCL10 does not differ between Pristane + PBS and Pristane + i.p. MTX groups in the acute and chronic phases of pristane-induced arthritis (*p* > 0.05). **b** P.a. MBV decreases serum concentration of CXCL10 in both the acute and chronic phase of pristane-induced arthritis (*p* < 0.05). **c** I.v. MBV decreases serum concentration of CXCL10 in both the acute and chronic phase of pristane-induced arthritis (*p* < 0.05). **d** Serum concentration of MCP-3 does not differ between Pristane + PBS and Pristane + i.p. MTX groups in the acute and chronic phases of pristane-induced arthritis (*p* > 0.05). **e** P.a. MBV decreases serum concentration of MCP-3 in both the acute and chronic phase of pristane-induced arthritis (*p* < 0.05). **f** I.v. MBV decreases serum concentration of MCP-3 in both the acute and chronic phase of pristane-induced arthritis (*p* < 0.05). All values in panels **a**–**f** represent mean ± s.e.m. (*n* = 3).

After disease induction on days 0 and 4, serum CXCL10 and MCP-3 concentrations in the Pristane + PBS group were significantly elevated compared to the Control + PBS group (Fig. 3a–f, *p* < 0.05). The pre-treatment CXCL10 and MCP-3 concentration spike (day 7) was observed in all groups and was significantly elevated compared to the Control + PBS group (Fig. 3a–f, *p* < .05). In the acute phase of the disease (day 0–42), serum concentration of CXCL10 and MCP-3 in the Pristane + PBS group declined between days 7 and 42 but always remained elevated compared to the Control + PBS group (Fig. 3a–f, *p* < 0.05). For both CXCL10 and MCP-3, Pristane + i.p. MTX was not significantly different from the Pristane + PBS throughout days 7–42 (Fig. 3a and d, *p* > 0.05) and was persistently elevated compared to Control + PBS (Fig. 3a and d, *p* < 0.05). Compared to Pristane + PBS, Pristane + p.a. MBV reduced serum CXCl10 concentrations at days 14 and 21 and serum MCP-3 at days 7–28 (Fig. 3b and e, *p* < 0.05). Compared to Pristane + PBS, Pristane + i.v. MBV reduced serum CXCL10 concentrations at days 14, 21, and 42 and reduced serum MCP-3 levels days 21–42 (Fig. 3c and f, *p* < 0.05). The Pristane + p.a. MBV group reduced CXCL10 concentration to levels equivalent to Control + PBS at day 21 (Fig. 3b, *p* > 0.05). The Pristane + i.v. MBV group reduced CXCL10 and MCP-3 to levels equivalent to the Control + PBS groups at days 14–42 and 21–42, respectively (Fig. 3c and f, *p* < 0.05).

In the chronic phase of disease (days 56–100), CXCL10 and MCP-3 concentrations in the Pristane + PBS group increased a second time on day 56 (Fig. 3. a–f). Likewise, similar to what was observed in the acute phase of disease, Pristane + i.p. MTX did not differ from Pristane + PBS and showed increased CXCLX10 and MCP-3 concentrations at day 56 (Fig. 3a and d, *p* > 0.05). Both the Pristane + p.a. MBV and Pristane + i.v. MBV did not have a CXCL10, or MCP-3 spike at day 56 and the concentrations of these chemokines was significantly reduced compared to Pristane + PBS (Fig. 3b, c, e, and f, *p* < 0.05).

### MBV decrease synovial cellular infiltration and promote a resolution of inflammation by decreasing the ratio of pro-inflammatory M1-like synovial macrophages to anti-inflammatory M2-like synovial macrophages

**Fig. 4:**
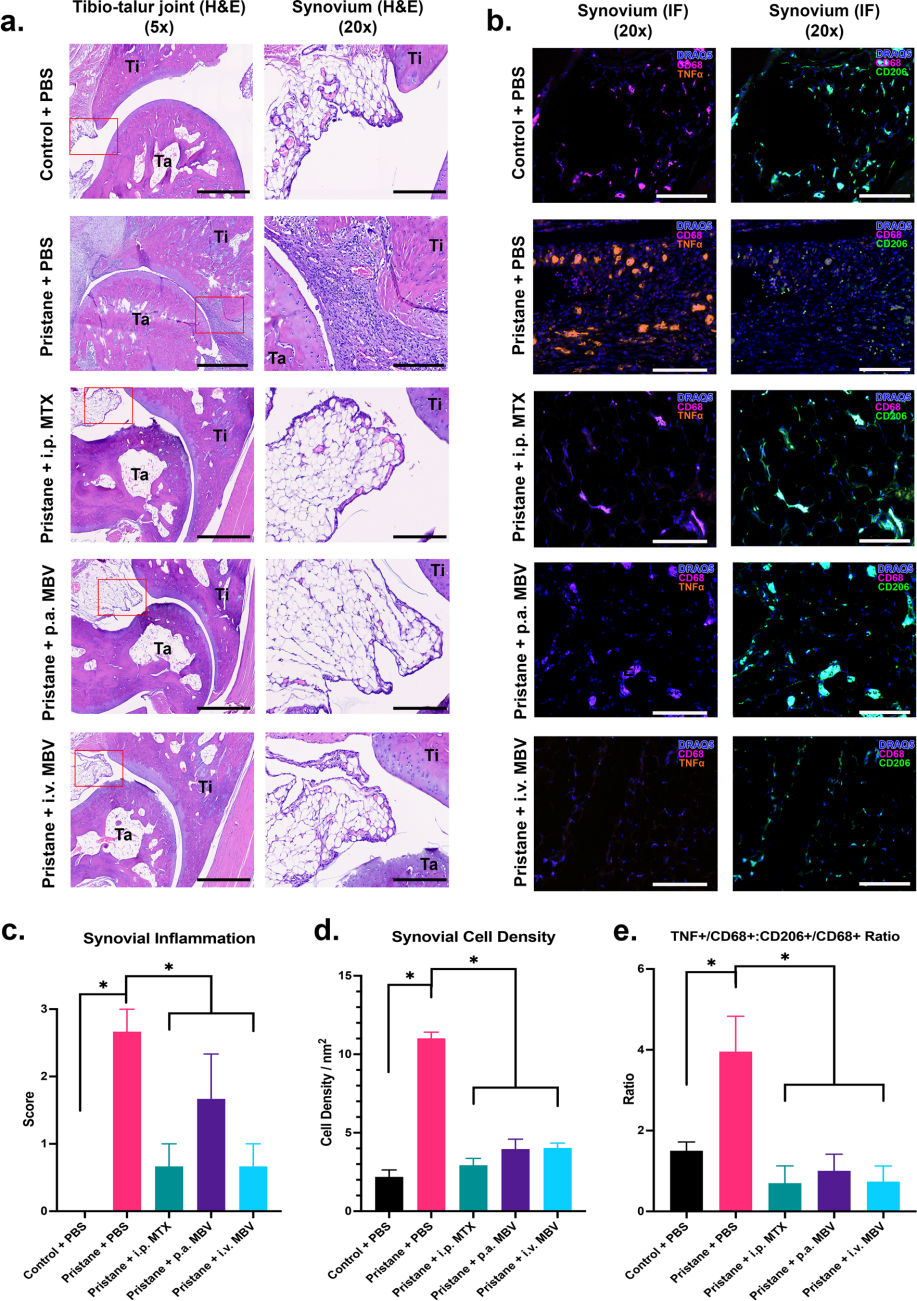
MBV reduce synovial inflammation and decrease the ratio of pro-inflammatory M1-like synovial macrophages to anti-inflammatory M2-like synovial macrophages. a Representative ×5 H&E images of the tibiotalar (tibia = Ti, talus = Ta) joint and ×20 H&E images of the adjacent synovium. Scale bar for ×5 images = 1000 µm. Scale bar for ×20 images = 200 µm. **b** ×20 immunofluorescent images of the synovium stained for M1-like synovial macrophages (DRAQ5+/CD68+/TNF-α+) in the left column and M2-like synovial macrophages (DRAQ5+/CD68+/CD206+) in the right column. Scale bar = 200 µm. **c** I.p. MTX, p.a. MBV, and i.v. MBV decrease overall synovial inflammation compared to the Pristane + PBS group (*p* < 0.05). **d** I.p. MTX, p.a. MBV, and i.v. MBV decrease synovial cellular density compared to the Pristane + PBS group (*p* < 0.05). **e** Compared to Control + PBS, pristane + PBS significantly increases the ratio of M1-like macrophages (TNF-α+/CD68+) compared to M2-like macrophages (CD206+/CD68+) (*p* < 0.05). All three treatment groups (i.p. MTX, p.a. MBV, and i.v. MBV) significantly reduce the ratio of M1-like:M2-like macrophages in the synovial tissue (*p* < 0.05) All values in panels **c**–**e** represent mean ± s.e.m. (*n* = 3).

Synovial inflammation, also called synovitis, has characteristic histologic features defined by immune cell infiltration. The comparative effects of MBV, MTX, and PBS treatment on synovial inflammation was determined by histopathologic examination of rat hindpaws at day 100. The vehicle-treated animals (Pristane + PBS group) developed joint pathology characterized by increased synovial cell infiltration with a dominant pro-inflammatory phenotype (Fig. 4a–e). The Pristane + PBS group showed a significant increase in synovial inflammation (2.7 ± 0.3 vs. 0.0 ± 0.0) when compared to Control + PBS group (Fig. 4a and c, *p* < 0.05). Compared to the Pristane + PBS group (2.7 ± 0.3), all treatment groups—Pristane + MTX (0.7 ± 0.3); p.a. MBV (1.7 ± 0.7); and i.v. MBV (0.7 ± 0.3)—showed reduced cellular infiltration of the synovium and reduced inflammation by semi-quantitative histologic scoring (Fig. 4a and c, *p* < 0.05). No differences were observed among the three treatment groups in semi-quantitative scoring of synovitis (Fig. 4a and c, *p* > 0.05). Synovial cellular infiltration was also quantified using an artificial intelligence assisted image analysis software (QuPath)^41^. The Pristane + PBS group showed significantly increased cellular density of the tibiotalar joint synovium compared to the control + PBS group (11.0 ± 0.4 cells/nm^2^ vs. 2.2 ± 0.4 cells/nm^2^) (Fig. 4a and d, *p* < 0.05). All three treatment groups—Pristane + MTX (2.9 ± 0.4 cells/nm^2^); p.a. MBV (3.9 ± 0.6 cells/nm^2^); and i.v. MBV (4.0 ± 0.3 cells/nm^2^)—significantly reduced synovial cellular infiltration compared to pristane + PBS (Fig. 4a and c, *p* < 0.05). There was no difference in cellular infiltration among the treatment groups and, collectively, between any of the treatment groups and the Control + PBS group (Fig. 4a and d, *p* > 0.05).

The disequilibrium between pro-inflammatory M1 macrophages and anti-inflammatory M2 macrophages is a key component of RA pathology. Compared to Control + PBS group, the Pristane + PBS group showed an increased ratio of synovial M1-like macrophages (TNF-α+/CD68+) relative to synovial M2-like macrophages (CD206+/CD68+) (3.9 ± 0.9 vs. 1.5 ± 0.2, Fig. 4b and e, *p* < 0.05). The ratio of M1-like:M2-like macrophages was significantly decreased in the Pristane + MTX group (0.7 ± 0.4, Fig. 4b and e, *p* < 0.05), Pristane + p.a. MBV group (1.0 ± 0.4, Fig. 4b and e, *p* < 0.05), and Pristane + i.v. MBV group (0.7 ± 0.4) when compared to the Pristane + PBS group (Fig. 4b and e, *p* < 0.05). There were no significant differences in the ratio of M1-like:M2-like macrophages among different treatment conditions; similarly, there were no significant differences between the treatment conditions and the Control + PBS group (Fig. 4b and e, *p* > 0.05).

### MBV decrease the ratio of splenic pro-inflammatory M1-like macrophages to anti-inflammatory M2-like macrophages and regulate a myeloid population shift driven by CD43hi/His48lo/CD206+ circulating monocytes

**Fig. 5:**
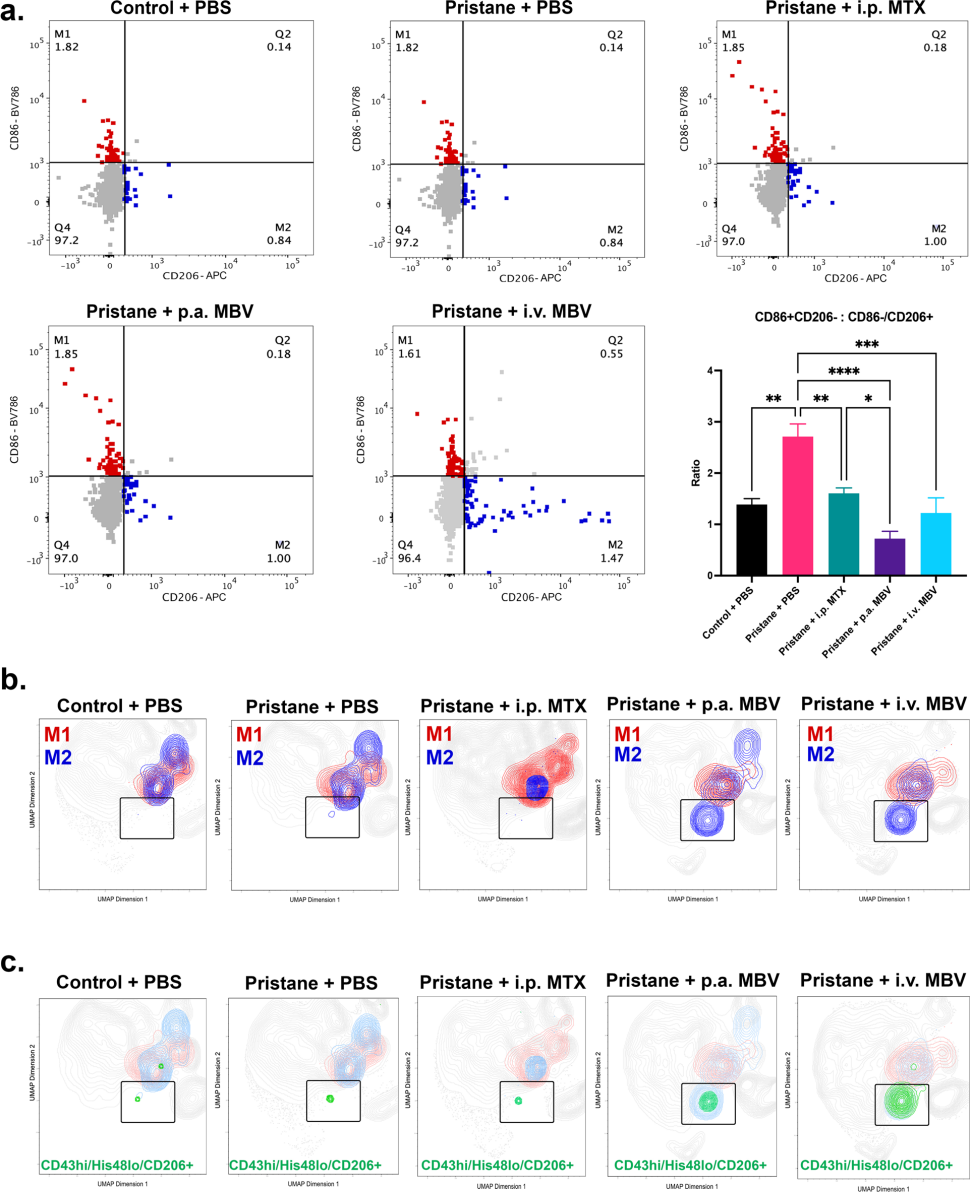
Local and systemic administration of MBV decrease the ratio of splenic pro-inflammatory M1-like macrophages to anti-inflammatory M2-like macrophages and regulates a myeloid population shift driven by CD43hi/His48lo/CD206+ circulating monocytes. **a** i.p. MTX, p.a. MBV, and i.v. MBV decrease the ratio of splenic pro-inflammatory, M1-like macrophages (CD68+/CD86+/CD206−) to anti-inflammatory, M2-like macrophages (CD68+/CD86−/CD206+) compared to Pristane + PBS (*p* < 0.05). **b** UMAP dimensional reduction of the entire splenic myeloid compartment revealed an M2-predominant cluster as outlined in rectangular outline that is unique to MBV treatment. **c** The M2-predominant cluster unique to MBV treatment is driven by immunoregulatory, CD43hi/His48lo/CD206+ circulating monocytes in the spleen. All values in panels **a** represent mean ± s.e.m. (*n* = 4). UMAP plots in **b** and **c** represent concatenated values of four biological replicates.

Systemic populations of pro-inflammatory M1-like and anti-inflammatory M2-like macrophages and monocytes were characterized by flow cytometry. The ratio of splenic pro-inflammatory M1-like macrophages (CD68+/CD86+/CD206−) to anti-inflammatory M2-like macrophages (CD68+/CD86−/CD206+) was significantly increased in the Pristane + PBS group compared to the Control + PBS group (Fig. 5a, *p* < 0.05). All three treatment groups (Pristane + i.p. MTX, Pristane + p.a. MBV, and Pristane + i.v. MBV) significantly reduced the ratio of M1-like:M2-like macrophages in the spleen (Fig. 5a, *p* < 0.05). All three treatment groups restored the splenic M1-like:M2-like macrophage ratio to that of the Control + PBS group (Fig. 5a, *p* > 0.05). To elucidate differences between treatment groups further, uniform manifold approximation and projection (UMAP) dimensional data reduction was performed on the entire myeloid population of the animal spleens. Using M1-like (CD68+/CD86+/CD206−) and M2-like (CD68+/CD86−/CD206+) gating strategies, the M1-like and M2-like population clusters were visualized in two dimensions (Fig. 5b). At study termination, there were no appreciable differences in population clustering between that of the Control + PBS and Pristane + PBS groups (Fig. 5b). However, all three of the treatment groups generated unique population maps of the M1-like and M2-like populations (Fig. 5b). Both the Pristane + p.a. MBV and Pristane + i.v. MBV generated a unique population of M2 cells that was not observed across the remaining groups (Fig. 5b outlined with a black rectangle). This population cluster was only found in MBV treatment groups. Further interrogation of this MBV-driven cluster revealed an enrichment in CD43hi/His48lo/CD206 + monocytes (Fig. 5c). As seen with the M1-like and M2-like populations, both the Pristane + p.a. MBV and Pristane + i.v. MBV groups were the only groups to show this strong CD43hi/His48lo/CD206+ clustering in the region of interest (Fig. 5c).

## Discussion

The results of the present study show that both systemic and local administration of porcine ECM MBV are as equally effective as MTX in mitigation of acute and chronic pristane-induced RA in the rat. Although all three treatments reduce synovial inflammation, cartilage destruction, and pro-inflammatory synovial and systemic macrophage phenotype, only the MBV treatment decreased clinically relevant pro-inflammatory serum chemokines and promoted a systemic myeloid shift towards an anti-inflammatory and immunoregulatory phenotype. Stated differently, MBV treatment had a distinctive and favorable immunomodulatory effect, and one that was different from that resulting from immunosuppressive MTX treatment. RA is a complex disease of multifactorial etiology and characterized by diverse and dysregulated interactions between immune and non-immune cells. While it is well understood that the imbalance of pro-inflammatory M1 and anti-inflammatory M2 macrophages contributes to disease pathophysiology, there are no available therapeutics directed selectively at resolving this disequilibrium.

Although the specific molecular mechanisms of action of MBV have yet to be determined, the favorable decrease in the ratio of M1-like:M2-like macrophages is a consistent and reproducible finding following ECM or MBV exposure. MBV have been shown in vitro to induce macrophage gene and protein expression patterns, cell surface markers, and functional capacities of anti-inflammatory, pro-remodeling M2-like macrophages^7,20^. The immunomodulatory effects of ECM and embedded MBV include increased phagocytic activity, decreased iNOS with decreased nitric oxide (NO) production, and increased antimicrobial activity, all of which are representative of an M2-like macrophage phenotype^5,6,10–13,16,42^. Preclinical and clinical studies have shown that ECM degradation products, including MBV, promote tissue regeneration and repair through the induction of an anti-inflammatory, pro-remodeling macrophage phenotype^1,4,5,7,10–13,18,19,42–52^. This ECM-induced macrophage phenotype regulation occurs while preserving the ability to mount a robust immune response to a variety of antigenic stimuli in a mouse model of antigenic challenge^47,49^. ECM biomaterials in a variety of formulations promote this favorable immunoregulatory effect in the context of volumetric muscle loss, topical wound healing, body wall repair, and of particular relevance to the present results, in a model of ulcerative colitis^8,12,45,51,53–56^. A recent study shows that intravitreal administration of MBV protected against ischemia-induced retinal ganglion cell axon degeneration and death, as well as preserved visual function in an in vivo rodent model of retinal damage^52^. Results from that same study showed that MBV prevented injury-induced decreases in growth associated protein-43 and injury-induced increases in glial fibrillary acidic protein through mitigation of pro-inflammatory signaling by activated microglia and astrocytes^52^. ECM and derivative MBV thus show consistent, reproducible, and constructive immunomodulatory effects in a variety of body systems and disease states.

Mitigation of the synovial inflammatory response with MTX occurs via immunosuppression, prevention of peripheral monocyte maturation, and decreased monocyte recruitment to the synovium^57^. MBV have identical effects to MTX on synovial cellular infiltration regardless of route of administration. However, unlike MTX treatment, both routes of MBV administration decrease CXCL10 and MCP-3, with both chemokines being clinically relevant markers of RA progression. In addition, both p.a. and i.v. MBV promote the development of a distinctive M2-like and CD43hi/His48lo/CD206+ systemic phenotype. CD43hi/His48lo monocytes in rat represent a population of circulating, immunoregulatory monocytes analogous to the well-characterized Ly6Clo murine monocytes^58–60^. These patrolling monocytes, also termed “nonclassical” monocytes, are distinguishable from “classical” monocytes by their ability to patrol tissue vasculature, to remove cellular debris, and can differentiate into M2-like, anti-inflammatory macrophage populations within inflamed tissues^59,61^. These CD43hi, nonclassical monocytes have been shown to participate in the resolution of joint inflammation in animal models of RA^61^. Since both p.a. and i.v. routes of MBV administration promote the development of these CD43hi/His48lo and CD206+(M2-like) monocyte population, the increased presence of these nonclassical CD43hi/His48lo/CD206+ monocytes may play an important role in the mitigation of pristane-induced arthritis observed in the present study.

It is likely that both the local and systemic administration of MBV ameliorate disease pathology through a combination of decreased recruitment of pro-inflammatory cell populations, a result consistent with the decreased chemokine secretion, and immunomodulation of systemic macrophage and monocyte precursor populations. The systemic administration route allows for MBV to interact with inflammatory cell populations upstream of their synovial involvement as, for example, with circulating and splenic monocytes and lymphocytes. In the pristane model, an intradermal administration of pristane induces disease pathology by interacting with monocytes and lymphocytes at the lymph nodes that drain the skin of the tail^62–64^. MBV administered by intravenous injection may interact at the site of peripheral lymphatics that drain intradermal pristane or interact at central lymphatic tissues such as the spleen or bone marrow. MBV administered at the peri-articular location may interact either directly with immune cells at the sight of the synovium, adjacent soft tissue, or with local immune cell populations that interact with further inflammatory mediators at central sites such as the lymph nodes or spleen^65^. Regardless of route of administration, treatment with MBV reduces pro-inflammatory mediators in pristane-induced arthritis and promotes the development of an immunoregulatory, systemic myeloid immune response. Further investigation, including biodistribution studies, are necessary to elucidate any differences between local and systemic administration of MBV.

While the present study describes the use of MBV therapy in a preclinical rat model of RA, the concept of using extracellular vesicles to treat autoimmune disease, specifically RA, is not new. Since 2005, various studies in animals and in humans have investigated the efficacy of fluid-phase extracellular vesicles (i.e., exosomes) in RA disease models and in patients with RA. These therapies have focused on genetic modification of bone-marrow-derived dendritic cells to produce genetically modified extracellular vesicles^66^. These engineered vesicles range from packaging exosomes with anti-inflammatory molecules such as IL-10^67,68^ and IL-4^69^, inducing exosome surface expression of pro-apoptotic molecules, such as FasL^70^ or immune checkpoint inhibitors such as CTLA4-Ig^71^, or isolating exosomes from plasma of antigen-immunized mice^72^. All such approaches have shown pre-clinical efficacy in RA through immunomodulation of innate and adaptive immune cell populations^67–72^. MBV, a distinct population of extracellular vesicles, represent an alternative to these fluid-phase exosome therapies with minimal processing and manipulation in comparison^20,73^.

The finding that both systemic and local MBV administration modulates synovial and systemic macrophage and monocyte phenotype from a M1-like-predominant, pro-inflammatory state towards an M2-like, anti-inflammatory state has broad implications for future RA studies. The M1-like:M2-like disequilibrium present in RA not only serves a role in the initial inflammation driving disease but contributes to the perpetuation of symptoms that lead to chronic disease. An aberrant predominance of M1-like macrophages targets both cells of the adaptive immune system and local mesenchymal cells of the joint in RA patients, specifically, CD4+ T-helper cells of the adaptive immune system and resident synovial fibroblasts in the joint^22,31,35^. In RA, there is a notable increase in circulating and joint-localized Th1 and Th17 cells, essentially creating a CD4 T-cell phenotype indicative of chronic inflammation and autoimmunity. Patients with RA also show a reduction in Th2 and Treg CD4+ T-helper cells^74^. The increase in pro-inflammatory and auto-immune associated CD4 T-cells is thought to be driven by local and systemic changes in the macrophage population that favor an M1-like state^75^. In contrast, disease remission and immunologic tolerance are characterized by a predominant Th2 and Treg phenotype^23,29–33,39,75–80^.

In addition to the immune cell component of disease pathology, fibroblasts present in the synovial parenchyma contribute to early-stage and late-stage findings of joint damage^81–89^. Pro-inflammatory M1-like macrophages in the joints of RA patients produce an abundance of TNFα and IL1β that directly stimulate the production of cytokines and matrix-metalloproteases (MMPs) by synovial fibroblasts that perpetuate inflammation and result in degradation of cartilage and bone^30,36,80,90–93^. Specifically, the M1-like macrophage secretome drives the production of synovial fibroblast-derived TNFα, Il-1, Il-6, and MMPs^94^. These pro-inflammatory cytokines and MMPs function in positive feedback to promote further inflammation and joint remodeling^25,80,81,89,90,95–98^. This lymphocytic and monocytic inflammation and fibroblast-derived adverse ECM remodeling results in the early-stage and late-stage clinical signs of joint pain and eventual joint destruction and functional impairment in RA.

While the results of the present study show the immunomodulatory potential of MBV in the pristane model of RA, the molecular mechanism(s) of immunomodulation are not fully understood. Additional studies are necessary to elucidate any potential differences in the mechanisms of action between local and systemic MBV administration and the mechanism by which enrichment of an M2-like, non-classical monocyte population in the myeloid immune system is achieved. The present study only included a single treatment regimen and dose of MBV. It is reasonable to assume that the present study used a suboptimal treatment strategy, and it is therefore possible that MBV technology can be improved.

The findings of the present study suggest that the immunomodulatory properties of ECM-based materials, specifically the MBV component of ECM-based materials, have therapeutic potential for diseases driven by a dysregulated immune system such as RA. The anti-inflammatory effects of ECM-based products have been well documented, and the expanded clinical applications made possible by MBV are worthy of further investigation.

## Methods

### Preparation of urinary bladder ECM

Urinary bladder ECM (UB-ECM) was prepared from market-weight pigs as previously described^9,73^. Porcine urinary bladders from market-weight animals (approximately 240 lbs.) were acquired from Animal Biotech Industries (Doylestown, PA, USA), and the tunica serosa, tunica muscularis externa, and most of the tunica submucosa and tunica muscularis mucosa of the bladders were mechanically removed. The luminal urothelial cells of the tunica mucosa were then dissociated from the basement membrane by washing with deionized water. The remaining tissue consisted of basement membrane, subjacent lamina propria of the tunica mucosa, and remnants of the tunica submucosa. This tissue was decellularized and digested by agitation in 0.1% peracetic acid with 4% ethanol for 2 h at 300 rpm. The tissue was then extensively rinsed with 1× PBS (pH 7.4) and sterile deionized water. Finally, the UB-ECM was lyophilized and milled into particulate using a Wiley mill (Thomas Scientific, Swedesboro, NJ, USA) with a #40 mesh screen.

### Isolation of MBV

MBV were isolated from laboratory produced porcine UB-ECM by enzymatic digestion^73^. Enzymatic digestion was performed using *Liberase* TL (Roche, Basel, Switzerland) (highly purified Collagenase I and Collagenase II) in buffer (50 mM Tris pH 7.5, 5 mM CaCl_2_, 150 mM NaCl) for 24 h at room temperature on an orbital rocker. Digested UB-ECM was then subjected to centrifugation at 10,000×*g* for 30 min at 4 °C and filtered through a 0.22-µm filter. The clarified supernatant containing the liberated MBV was then centrifuged at 100,000×*g* (Optima L-90K Ultracentrifuge, Beckman Coulter, Brea, CA, USA) at 4 °C for 70 min to pellet the MBV. MBV were then re-suspended in sterile 1× PBS (pH 7.4), and particle concentration was determined using particle nanotracking analysis.

### Pristane-induced arthritis and matrix-bound nanovesicle therapy

Inflammatory arthritis was induced in 8-week-old, female, Sprague-Dawley rats by an intradermal injection with 300 µL of pristane (2,6,10,14-tetramethypentadecane) at the dorsal side of the tail, 1 cm distal to the base on day 0 of the study, as previously described^62,64,99^. A second dose of 300 µL pristane was administered intra-dermally, approximately 1 cm distal to the dorsal tail base on day 4. Control + PBS animals did not receive an intradermal injection of pristane on day 0 and day 4. Animals receiving pristane were then randomized into four groups: Pristane + PBS; Pristane + intraperitoneal (i.p.) Methotrexate (MTX); Pristane + peri-articular (p.a.) MBV; and Pristane + intravenous (i.v.) MBV. The severity of arthritis was scored for each animal on days 7, 10, 14, 17, 21, 28 and weekly thereafter through day 100. Qualitative arthritis severity score was evaluated by two independent reviewers using the following 60-point arthritis scoring criteria: 1 point was given for each inflamed proximal-interphalangeal joint or distal-interphalangeal joint, and up to 5 points were assigned for an affected ankle or wrist (Maximum score = 15 points per paw, 60 points per each rat)^64^. Animals designated as Pristane + PBS did not receive any treatment on days 7, 10, 14, 17, and 21. The pristane + i.p. MTX animals received 0.1 mg/kg MTX in sterile 1× PBS (pH 7.4) delivered i.p. on days 7, 10, 14, 17, and 21. The Pristane + p.a. MBV animals received 25 µL of 1 × 10^11^ MBV particles/mL, delivered in the plantar and volar surfaces of each hind paw and forepaw, respectively. The Pristane + i.v. MBV group received 100 µL of 1 × 10^11^ MBV particles/mL via the lateral tail vein on days 7, 10, 14, 17, and 21 (Fig. 1a). All animal work conducted in the present study was approved by the University of Pittsburgh Institutional Animal Care and Use Committee (Protocol number: 18103654).

### Histopathology and immunohistochemistry

One hind paw from each animal was used for histopathological analysis of the tibiotalar. Tissue specimens were fixed in 10% neutral-buffered formalin, pH 7.4, decalcified using 5% formic acid, and embedded in paraffin wax. Sections were stained with hematoxylin and eosin (H&E) for examination by light microscopy. Slides were imaged using a MoticEasyScan (Schertz, TX, USA) digital slide scanner. Inflammation was evaluated by an adapted semi-quantitative scoring system^100^. Inflammation was scored on a scale of 0–3 (with 0 representing no inflammation and 3 a severely inflamed joint) depending on the number of inflammatory cells in the synovial tissue. Quantitative determination of cellular infiltration was performed using QuPath^41^.

Macrophage phenotype in the synovial tissue adjacent to the tibiotalar joint was assessed by immunolabeling. Paraffin-embedded tissue sections were deparaffinized using three progressive washes of xylene, followed by rehydration using decreasing ethanol exchanges from 100% to 70% ethanol. Antigen retrieval was performed using commercially available DeCal solution per manufacturer’s protocol (BioGenex). Sections were then blocked for one hour at room temperature using 5% bovine-serum albumin in 1× tris-buffered saline, pH 7.4. Sections were incubated ~18 h at 4 °C with primary antibodies and respective dilutions (Supplementary Table 1). Following primary antibody incubation, sections were incubated for 1 h at room temperature with the following fluorescently conjugated secondary antibodies (Supplementary Table 2). Stained slides were imaged using Zeiss Axio-observer Z1 microscope. M1-like macrophages were identified by dual-positive staining with TNF-α and CD68, and M2-like macrophages were identified by dual-positive staining with CD206 and CD68. Dual positive staining was assessed using Cell Profiler software^101^.

### micro-Computed Tomography (microCT) bone assessment

Rat forepaws and hind paws including tibiotalar joints were collected at euthanasia and fixed in 10% formalin in PBS, pH 7.4, for 48 h. microCT scans were performed to evaluate bone morphology and architecture using Viva-CT 40 (SCANCO Medical AG, Bruttisellen, Switzerland) with the following settings: energy 70 kV, intensity 114 µA; integration time 300 ms; and isotropic voxel size of 10.5 µm. 3-D reconstruction was carried out using the acquired 2-D lateral projections using VivaCT40 operating software interface.

### Serum cytokine and chemokine assessment

Blood was collected from the tail vein of animals once weekly from days 0–100. Serum cytokine and chemokine concentrations were assessed using rat-specific, bead-based, multiplex ELISA (Thermo Fisher Scientific, EPX220-30122-901). Sample assay was performed in duplicate.

### Multiparameter flow cytometry

Multiparameter flow cytometry was performed on splenocytes collected at study termination (day 100). Briefly, spleens were minced in ice-cold 10% v/v FBS/PBS with scissors. Red blood cell lysis was performed using ammonium chloride. Single cell suspension of splenocytes were stained with a fixable viability dye (1:1000, FVDe506, eBioscience 65-0866-18) for 30 min on ice. After viability staining, cells were stained with an extracellular surface marker cocktail for 30 min on ice containing the fluorescently conjugated antibodies at their respective dilutions (Supplementary Table 3). Cellular fixation and permeabilization were performed for 1 h and intracellular antigens were stained for 1 h with fluorescently conjugated antibodies (Supplementary Table 3). Compensation beads were stained at working antibody concentrations and used for all fluorescent compensation to establish gating criteria (Supplementary Fig. 1). Stained samples were analyzed using a BD FACSaria^TM^ II flow cytometer and results were analyzed using FlowJo v10 (BD, Franklin Lakes, NJ, USA). Uniform Manifold Approximation and Projection (UMAP) dimensional reduction was performed using FlowJo v10 with the following parameters: Euclidean; nearest neighbors = 15; minimum distance = 0.5; and number of components = 2^102^.

### Statistical analysis

Sample size was determined using previously published effect size of methotrexate with a predetermined alpha 0.05 and power 0.80^64^. Significant differences were determined as *p* **<** 0.05. Arthritis score was represented as mean ± standard error of mean. Histology and bone erosion scores are represented as mean ± standard error. Serum data represents an *n* of 3 and values are reported as mean ± standard error. Flow cytometry data represent an *n* of 4 and values are reported as mean ± standard error. Days 7–21 included an *n* of 12 for each group and then day 28 and onward included an *n* of 8 for each group. Differences among groups were determined using two-way analysis of variance with Tukey’s post-hoc correction with no adjustments made for multiplicity. All data were analyzed using Prism 9 (GraphPad).

### Reporting summary

Further information on research design is available in the Nature Research Reporting Summary linked to this article.

## Supplementary information

Supplemental Figures and Tables

REPORTING SUMMARY

## Data Availability

The data that support the findings of this study are available from the corresponding author upon request.
