# Supplementary material for: Immunomodulatory matrix-bound nanovesicles mitigate acute and chronic pristane-induced rheumatoid arthritis
Source: NPJ Regen Med. 2022 Feb 2;7:13. doi: 10.1038/s41536-022-00208-9 (PMC8810774; doi:10.1038/s41536-022-00208-9)
Supplement: Supplementary file 1 — Supplemental Figures and Tables [file 41536_2022_208_MOESM1_ESM.pdf]

Supplemental Data and Information

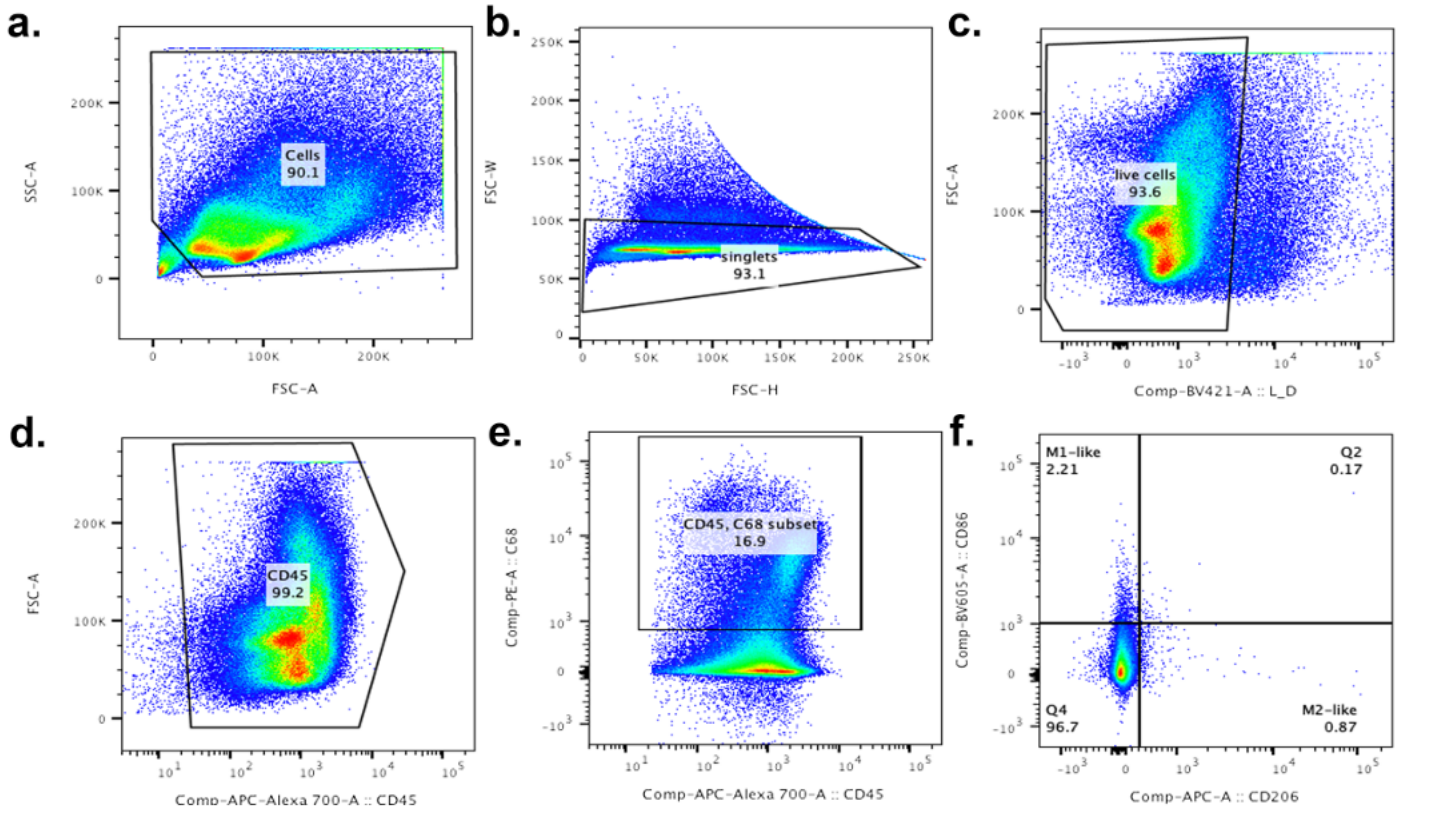

Supplementary Figure 1. Gating strategy of flow cytometry analysis. a.) Total events with exclusion of smaller cells like red-blood cells. b.) Doublet exclusion. c.) Selection of live cells. d.) Selection of CD45+ cells. e.) Selection of CD45+/CD68+ cells. f.) Selection of M1-like and M2-like cells by gating on CD86 (M1-like) and CD206 (M2-like) expression.

Supplementary Table 1. Primary antibodies used in immunohistochemistry

Target	Host	Clone/ID	Vendor	Cat #	Dilution
DRAQ5	NA	NA	Thermo Scientific	PI62251	1:1000
CD68	Goat	ED1	Bio-Rad	MCA341GA	1:100
TNF-alpha	Rabbit	Polyclonal	Abcam	Ab6671	1:100
CD206	Mouse	Polyclonal	R&D Systems	AF2535	1:100

Supplementary Table 2. Secondary antibodies used in immunohistochemistry

Target	Host	Conjugate	Vendor	Cat #	Dilution
Rabbit	Donkey	AF350	Thermo Scientific	A10039	1:500
Mouse	Goat	AF488	Invitrogen	A11029	1:500
Goat	Donkey	AF594	Thermo Fisher	A-11058	1:500

Supplementary Table 3. Antibodies used in flow cytometry

Targets	Conjugate	Vendor	Cat #	Clone	Lot	Dilution
Viability Dye	e506	eBioscience	65-0866-18	NA	NA	1:1000
CD32	---	BD	550271	D34-485	0072019	1:200
CD45	AF700	Biolegend	202218	OX-1	B346320	1:100
CD43	PE/Cy7	Biolegend	202816	W3/13	B312623	1:200
His48	FITC	BD	554907	HIS48	1140679	1:200
CD161a	BV650	BD	744052	10/78	1250125	1:200
CD86	BV786	BD	743216	24F	1250715	1:50
CD206	APC	BD	550889	19.2	1103032	1:20
CD68	PE	Thermo Scientific	MA5-16653	ED1	WI3376801	1:20
