# Supplementary material for: Immunomodulatory matrix-bound nanovesicles mitigate acute and chronic pristane-induced rheumatoid arthritis
Source: NPJ Regen Med. 2022 Feb 2;7:13. doi: 10.1038/s41536-022-00208-9 (PMC8810774; doi:10.1038/s41536-022-00208-9)
Supplement: Supplementary file 2 — REPORTING SUMMARY [file 41536_2022_208_MOESM2_ESM.pdf]

nature portfolio

Corresponding author(s): DBPR; NPJREGENMED-00581R

Last updated by author(s): Nov 17, 2021

Reporting Summary

Nature Portfolio wishes to improve the reproducibility of the work that we publish. This form provides structure for consistency and transparency in reporting. For further information on Nature Portfolio policies, see our Editorial Policies and the Editorial Policy Checklist.

Statistics

For all statistical analyses, confirm that the following items are present in the figure legend, table legend, main text, or Methods section.

n/a	Confirmed	
☐	☒	The exact sample size (*n*) for each experimental group/condition, given as a discrete number and unit of measurement
☐	☒	A statement on whether measurements were taken from distinct samples or whether the same sample was measured repeatedly
☐	☒	The statistical test(s) used AND whether they are one- or two-sided<br>*Only common tests should be described solely by name; describe more complex techniques in the Methods section.*
☒	☐	A description of all covariates tested
☐	☒	A description of any assumptions or corrections, such as tests of normality and adjustment for multiple comparisons
☐	☒	A full description of the statistical parameters including central tendency (e.g. means) or other basic estimates (e.g. regression coefficient) AND variation (e.g. standard deviation) or associated estimates of uncertainty (e.g. confidence intervals)
☐	☒	For null hypothesis testing, the test statistic (e.g. *F*, *t*, *r*) with confidence intervals, effect sizes, degrees of freedom and *P* value noted<br>*Give P values as exact values whenever suitable.*
☒	☐	For Bayesian analysis, information on the choice of priors and Markov chain Monte Carlo settings
☒	☐	For hierarchical and complex designs, identification of the appropriate level for tests and full reporting of outcomes
☒	☐	Estimates of effect sizes (e.g. Cohen's *d*, Pearson's *r*), indicating how they were calculated

Our web collection on statistics for biologists contains articles on many of the points above.

Software and code

Policy information about availability of computer code

Data collection	All data were collected and stored in Prism 9 (GraphPad).
Data analysis	Data were analyzed using Prism 9 (GraphPad). Flow cytometry data was analyzed using FlowJo v10 (BD, Franklin Lakes, NJ, USA).

For manuscripts utilizing custom algorithms or software that are central to the research but not yet described in published literature, software must be made available to editors and reviewers. We strongly encourage code deposition in a community repository (e.g. GitHub). See the Nature Portfolio guidelines for submitting code & software for further information.

Data

Policy information about availability of data

All manuscripts must include a data availability statement. This statement should provide the following information, where applicable:

- Accession codes, unique identifiers, or web links for publicly available datasets
- A description of any restrictions on data availability
- For clinical datasets or third party data, please ensure that the statement adheres to our policy

The data that support the findings of this study are available from the corresponding author upon request.

Field-specific reporting

Please select the one below that is the best fit for your research. If you are not sure, read the appropriate sections before making your selection.

☒ Life sciences ☐ Behavioural & social sciences ☐ Ecological, evolutionary & environmental sciences

For a reference copy of the document with all sections, see nature.com/documents/nr-reporting-summary-flat.pdf

Life sciences study design

All studies must disclose on these points even when the disclosure is negative.

Sample size	Sample size was determined using previously published effect size of methotrexate with a predetermined alpha 0l05 and power 0.2. Sample size calculations were performed using G*Power software.
Data exclusions	No data were excluded from the analyses.
Replication	All attempts at replication were successful in the present work.
Randomization	Animals were randomized to treatment groups.
Blinding	Investigators were blinded to treatment groups throughout the course of the animal study as well as during the scoring of the histology samples.

Reporting for specific materials, systems and methods

We require information from authors about some types of materials, experimental systems and methods used in many studies. Here, indicate whether each material, system or method listed is relevant to your study. If you are not sure if a list item applies to your research, read the appropriate section before selecting a response.

Materials & experimental systems

n/a	Involved in the study	
☐	☒	Antibodies
☒	☐	Eukaryotic cell lines
☒	☐	Palaeontology and archaeology
☐	☒	Animals and other organisms
☒	☐	Human research participants
☒	☐	Clinical data
☒	☐	Dual use research of concern

Methods

n/a	Involved in the study	
☒	☐	ChIP-seq
☐	☒	Flow cytometry
☒	☐	MRI-based neuroimaging

Antibodies

Antibodies used

Primary antibodies used for immunohistochemistry: CD68 (Host: goat; Clone: ED1, Vendor: Thermo Scientific PI62251), TNF-alpha (host: goat, Clone: Polyclonal, Vendor: Abcam AB6671), CD206 (Host:mouse, Clone: polyclonal, Vendor: R&D Systems AF2535) Secondary Antibodies used in immunohistochemistry: anti-rabbit AF350 (host: donkey, Vendor: Thermo Scientific A10039), anti-mouse AF488 (Host: goat, Vendor: Invitrogen A11029), anti-goat AF594 (Host: donkey, Vendor: Thermo Fisher A11058).

Antibodies used in flow cytometry:
Viability Dye (e506), Vendor: eBioscience 65-0866-18
anti-CD32 (unconjugated), Vendor: BD 550271, Clone: D34-485, Lot: 0072019
Anti-CD45 (AF700), Vendor: Biolegend 202218, Clone: OX-1, Lot: B346320
Anti-CD43 (PE/Cy7), Vendor: Biolegend 202816, Clone W3/13, Lot: B312623
Anti-His48 (FITC), Vendor: BD 554907, Clone: HIS48, Lot: 1140679
Anti-CD161a (BV650), Vendor: BD 744052, Clone: 10/78, Lot: 1250125
Anti-CD86 (BV786), Vendor: BD 743216, Clone: 24F, Lot: 1250715
Anti-CD206 (APC), Vendor: BD 550889, Clone: 19.2, Lot: 1103032
Anti-CD68 (PE), Vendor: Thermo Scientific MA5-16653, Clone: ED1, Lot: WI3376801

Validation

Validation data present in manufacturers' provided data sheets.

Animals and other organisms

Policy information about studies involving animals; ARRIVE guidelines recommended for reporting animal research

Laboratory animals	8 week-old, Female, Sprague-Dawley rats were used for the present study.
Wild animals	NA
Field-collected samples	NA
Ethics oversight	The animal study approval and oversight was through the University of Pittsburgh Institutional Animal Care and Use Committee (Protocol # 18103654)

Note that full information on the approval of the study protocol must also be provided in the manuscript.

Flow Cytometry

Plots

Confirm that:

☒ The axis labels state the marker and fluorochrome used (e.g. CD4-FITC).

☒ The axis scales are clearly visible. Include numbers along axes only for bottom left plot of group (a 'group' is an analysis of identical markers).

☒ All plots are contour plots with outliers or pseudocolor plots.

☒ A numerical value for number of cells or percentage (with statistics) is provided.

Methodology

Sample preparation	Briefly, spleens were minced in ice-cold 10% v/v FBS/PBS with scissors. Red blood cell lysis was performed using ammonium chloride. Single cell suspension of splenocytes were stained with a fixable viability dye (1:1000 (FVDe506, eBioscience 65-0866-18)) for 30 minutes on ice. After viability staining, cells were stained with an extracellular surface marker cocktail for 30 minutes on ice containing the fluorescently conjugated antibodies at their respective dilutions (Supplementary Table 3.). Cellular fixation and permeabilization were performed for 1 hour and intracellular antigens were stained for 1 hour with fluorescently conjugated antibodies (Supplementary Table 3.). Compensation beads were stained at working antibody concentrations and used for all fluorescent compensation to establish gating criteria (Supplementary Figure 1.).
Instrument	BD FACSariaTM II
Software	FlowJo v10 (BD, Franklin Lakes, NJ, USA).
Cell population abundance	The CD45+/CD68+ population of interest represent approximately 15-25% of the parent splenocytes isolated (~1500-2500 cells).
Gating strategy	Compensation beads were stained at working antibody concentrations and used for all fluorescent compensation to establish gating criteria (Supplementary Figure 1.).

☒ Tick this box to confirm that a figure exemplifying the gating strategy is provided in the Supplementary Information.
